# Retraction: Graphene oxide/DNA-decorated electrode for the fabrication of microRNA biosensor

**DOI:** 10.1039/d0ra90077j

**Published:** 2020-07-21

**Authors:** Erlin Sun, Lining Wang, Xiaodong Zhou, Chengquan Ma, Yan Sun, Mingde Lei, Bingxin Lu, Ruifa Han

**Affiliations:** Tianjin Institute of Urology, Department of Urology, The 2nd Hospital of Tianjin Medical University Tianjin 300211 P. R. China erlinsuntj@126.com +86 22 88326188

## Abstract

Retraction of ‘Graphene oxide/DNA-decorated electrode for the fabrication of microRNA biosensor’ by Erlin Sun *et al.*, *RSC Adv.*, 2015, **5**, 69334–69338, DOI: 10.1039/C5RA12373A.

We, the named authors, hereby wholly retract this *RSC Advances* article due to a lack of reproducibility in the results of the paper. We have been unable to replicate the results of miRNA detection, shown in Fig. 8, and as a result, the conclusions of this paper are not supported.

Signed: Erlin Sun, Lining Wang, Xiaodong Zhou, Chengquan Ma, Yan Sun, Mingde Lei, Bingxin Lu and Ruifa Han

Date: 14^th^ July 2020

Retraction endorsed by Laura Fisher, Executive Editor, *RSC Advances*

## Supplementary Material

